# Implications of Gene Therapy in Dentistry and Periodontics: A Narrative Review

**DOI:** 10.7759/cureus.49437

**Published:** 2023-11-26

**Authors:** Arpit Barhate, Pavan Bajaj, Unnati Shirbhate, Amit Reche, Abhishek Pahade, Ritiksha Agrawal

**Affiliations:** 1 Department of Dentistry, Sharad Pawar Dental College, Datta Meghe Institute of Higher Education and Research, Wardha, IND; 2 Department of Periodontics, Sharad Pawar Dental College, Datta Meghe Institute of Higher Education and Research, Wardha, IND; 3 Department of Public Health Dentistry, Sharad Pawar Dental College, Datta Meghe Institute of Higher Education and Research, Wardha, IND

**Keywords:** tissue repair and regeneration, gene transfer, periodontics, dentistry, vector, gene therapy

## Abstract

The relentless march of technological progress entails constant evolution and adaptation. A concerted effort is underway in medical research to unravel various diseases' cellular and molecular underpinnings. The traditional approaches to disease treatment often fall short of delivering entirely satisfactory outcomes, which has prompted a shifting spotlight on gene therapy as a versatile solution for many inherited and acquired disorders. Genes, intricate sequences of genetic code, are the complicated blueprints dictating the production of essential proteins within the human body. Remarkably, each individual's genetic makeup is uniquely distinct, with variations in these genetic sequences serving as the bedrock of our diversity. Gene therapy represents an innovative medical strategy that harnesses the power of genes themselves to function as therapeutic agents. It serves as a conduit through which defective genes are either substituted or mended with the introduction of remedial genetic material.

This groundbreaking method can tackle various illnesses, from conditions originating from single-gene abnormalities to intricate disorders influenced by multiple genes. In dentistry and periodontics, gene therapy finds a promising array of applications. It contributes significantly to managing salivary gland disorders, autoimmune diseases, and the regeneration of damaged bone tissue, as well as addressing cancerous and precancerous conditions. Moreover, the possibilities extend into DNA vaccination and broader areas of oral health. The advent of gene therapy in dentistry represents a new era of significant progress, offering substantial advancements in the management of periodontal disease and the reconstruction of the dental alveolar apparatus. The aim of this narrative review is to provide a comprehensive overview of the landscape of gene therapy investigations in these disciplines, shedding light on its potential implications for oral health and treatment. With its potential to rectify the genetic underpinnings of various conditions, gene therapy offers a novel frontier in healthcare that continually shapes the landscape of medicine and holds the promise of more effective and personalised treatments.

## Introduction and background

Gene therapy stands as a revolutionary domain within biomedicine, driven by the mission to rectify or replace flawed genes in the genomes of ailing cells, with a paramount objective of restoring normal cellular function while maintaining the utmost precision to prevent any harm to non-targeted tissues. Human gene therapy began its pioneering journey in 1980, beginning a relentless journey of scientific exploration and clinical trials [1,2]. Two primary categories exist in gene therapy: somatic gene therapy and germline gene therapy. Somatic gene therapy involves altering genes in specialised cells, excluding gametes or undifferentiated cells. This type of gene therapy results in genetic alterations that remain limited to the treated individuals and are not passed on to future generations. On the other hand, germ-line gene therapy aims to make enduring and heritable changes to an individual's genetic makeup by targeting their reproductive cells. However, due to ethical and technical complexities, most gene therapy research predominantly focuses on somatic cell gene therapy [3].

In gene therapy, the fundamental process often begins with discovering, isolating, and replicating therapeutic genes. These genes are then incorporated into a carrier called a vector. Vectors play a crucial role in ferrying the desired gene to the specific target tissue. Their primary function is to accurately transport the therapeutic genetic material into each target cell, thereby guaranteeing the success of the treatment. Viral and non-viral vectors serve as the primary categories for these vehicles. Viruses, renowned for their efficiency, are powerful tools for gene transfer. Commonly utilised viral vectors encompass retroviruses, adenoviruses, adeno-associated viruses, and herpes viruses. Furthermore, non-viral vectors contain a wide array of methodologies, including electroporation, microinjection, the utilisation of ballistic particles, calcium vectors, lipid vectors, and the creation of protein complexes [2,3]. This versatile array of vector options allows researchers to tailor their approach to the specific needs of each therapeutic endeavour [3].

## Review

The landscape of gene therapy in dentistry has witnessed remarkable strides since 1995 [4]. Yet, when we turn our gaze specifically towards periodontics, we find a more complex and challenging scenario. The successful application of gene therapy in periodontics has remained elusive primarily due to the inherent imperfections and substantial hurdles in gene therapy technology [5]. However, the enigma of periodontal disease further complicates matters. This ailment is a multifaceted entity arising from a confluence of factors, including microbial challenges and the highly variable responses of the host's immune system, which are further influenced by genetics and environmental conditions [6-8]. To further complicate matters, genetic variations arise within diverse populations, entailing intricate interactions among multiple genes and complex interchange between genes and environmental factors. No universally acknowledged single gene can be definitively attributed as a contributing factor for periodontal disease [9]. While initial ventures have been made to harness gene therapy tools to address periodontally relevant issues, they are still nascent, mainly existing in theory. Although certain preliminary animal model studies have shown encouraging outcomes, the practical implementation of gene therapy for periodontal diseases remains an enticing yet formidable objective that demands extensive research and development efforts [8,9].

History

Ever since the initiation of gene therapy research, a predominant belief has persisted among scientists that addressing inherited diseases at the genetic level holds the key to groundbreaking treatment possibilities, and the evolving comprehension of genetics, coupled with innovative techniques, has propelled us into the realm of contemporary gene therapy [10]. Table 1 explores pivotal moments in the progression of genetic research and the development of gene therapy.

**Table 1:** Genetic research and gene therapy timeline. [11-25]

Year	Pivotal moments in the progression of genetic research and the development of gene therapy
1953	The revelation of the DNA structure unveiled its iconic double helical configuration.
1973	The breakthrough in genetic engineering marked the ability to replicate and express DNA or RNA in different organisms, a pivotal discovery in genetic research.
1980	Ethical concerns were raised when a University of California at Los Angeles, United States (US), researcher conducted unauthorised gene therapy experiments on humans. This resulted in the revocation of multiple research grants and a stern warning from the National Institutes of Health (NIH) against future unauthorised human experimentation.
1990	The inaugural clinical trial featuring viral vector technology marked a groundbreaking moment when two patients afflicted with the rare pediatric disorder known as severe combined immunodeficiency (SCID) became the recipients of pioneering treatment employing innovative gamma-retroviral vector technology.
1996	In a significant stride forward, researchers engineered the initial generation of lentiviral vectors (LVVs) derived from HIV. Subsequently, second and third generations of LVVs emerged as further technological advancements.
1999	As a response to a tragic incident involving the untimely demise of an 18-year-old patient, Jesse Gelsinger, during a clinical trial employing an adenoviral vector, both the US FDA and the NIH instituted novel programs aimed at bolstering safety measures and enhancing the transparency of gene therapy clinical trials.
2000	The field of gene therapy encountered another setback when ten patients with SCID were subjected to gene therapy utilizing gamma-retroviral vectors. This approach, unfortunately, led to the development of leukaemia in four of the treated individuals, prompting grave concerns regarding the safety of gene insertion and emphasising the imperative of refining safety measures and mitigating toxicity associated with the use of viral vectors.
2003	A pivotal milestone in gene therapy was reached when the China FDA granted regulatory approval for the world's inaugural commercially available gene therapy designed to combat squamous cell carcinoma, a type of skin cancer.
2012	A historic moment unfolded as the European Medicines Agency (EMA) granted its first-ever approval for an in vivo gene addition therapy utilising adeno-associated virus (AAV) vectors, intended to address a rare inherited condition known as lipoprotein lipase deficiency (LPLD). In a momentous leap forward, scientists introduced a cutting-edge gene-editing method known as clustered regularly interspaced short palindromic repeats (CRISPR-Cas9), which carries the transformative capability to precisely modify specific segments of DNA sequences.
2016	A groundbreaking achievement in the European Union's regulatory landscape occurred as the EMA granted its first-ever ex vivo gene therapy approval. Specifically, this approval pertains to a gamma-retroviral vector-based gene addition therapy aimed at treating adenosine deaminase deficient SCID (ADA-SCID).
2017	A momentous milestone unfolded as the US FDA granted its inaugural approval for an in vivo gene addition therapy. This groundbreaking therapy employs AAV vectors and addresses a genetic eye ailment leading to progressive vision loss.
2018	In a pioneering clinical endeavour, researchers embarked on the first-ever clinical trial utilising CRISPR/Cas9 technology. This trial focused on harnessing CRISPR/Cas9 for gene disruption, explicitly targeting patients afflicted with genetic β-hemoglobinopathies.

As of today, the US FDA has received an impressive tally of over 900 applications for initiating clinical trials exploring gene therapy. This transformative field is steadily progressing from its research phase towards the prospect of regulatory approvals, all in pursuit of the noble aim of providing innovative therapeutic solutions for patient populations who benefit from these cutting-edge interventions [26].

Fundamentals of gene therapy

Gene therapy employs diverse techniques to replace or repair targeted genes. Every method is customised to meet the individual requirements of the patient and the specific genetic disorder under consideration. Gene insertion, homologous recombination, selective reverse mutation, gene regulation, and spindle transfer are the techniques to replace or repair targeted genes.

Gene Insertion

This is one of the most widespread strategies in gene therapy. This process entails inserting a healthy gene into the genetic code, which can be done at a designated or unspecified site in the genome. The goal is to substitute a dysfunctional or altered gene with one that functions correctly. This method seeks to restore the correct functioning of the gene and, consequently, the production of the required protein. It is widely used in gene therapy for various genetic disorders [27-29].

Homologous Recombination

In cases where an abnormal gene needs correction, homologous recombination is employed. This technique replaces the faulty gene with a healthy, regular counterpart. Its operation involves the smooth integration of the healthy gene into the genome, ensuring its seamless incorporation and the genetic material is appropriately replaced, thus restoring proper gene function. Homologous recombination is particularly valuable in addressing genetic disorders resulting from specific mutations [27-29].

Selective Reverse Mutation

Selective reverse mutation is used to correct abnormal genes. It involves a targeted reversal of modifications to restore the gene's normal function. By reverting the gene's sequence to its non-mutated state, this approach aims to rectify any genetic defects and promote the production of the required functional protein [27-29].

Gene Regulation

Gene therapy isn't limited to gene replacement or correction; it also includes techniques for fine-tuning gene activity. The regulation of a specific gene involves controlling the extent to which it is turned on or off. This precise control allows for the management of gene expression, ensuring that the gene functions optimally in response to the patient's needs. This can be especially valuable in treating conditions where overexpression or underexpression of a gene is a contributing factor [27-29].

Spindle Transfer

Spindle transfer is a unique and specialised technique for addressing mitochondrial disorders. It involves the replacement of entire mitochondria carrying defective mitochondrial DNA with healthy mitochondria. This is achieved by transferring the nuclear material (containing most of a cell's genetic information) from an egg or embryo with healthy mitochondria to the patient's egg or embryo. Spindle transfer offers a distinctive approach to treating conditions related to mitochondrial DNA mutations. It has been employed to aid in averting the transfer of mitochondrial diseases from mother to offspring [27,29].

Types of gene therapies

Germ-Line Gene Therapy

Gene therapy can be broadly classified into two primary categories with distinct implications and applications: germ-line gene therapy and somatic gene therapy. In germ-line gene therapy, modification of germ cells, including sperm and eggs, is involved. This approach introduces functional genes into these germ cells, and the cell's genetic material is typically incorporated with these therapeutic genes. What sets germ-line gene therapy apart is that any genetic changes instigated by this therapy are heritable. This implies that they can be transmitted to future generations. This heritability is both the strength and challenge of germ-line gene therapy. It provides the opportunity to address genetic disorders at their root, potentially eradicating them from a family's genetic lineage. However, it also carries significant ethical and safety concerns. Alterations to the human germline could have unforeseen consequences for future generations, and any errors or unintended effects could have long-lasting and far-reaching implications. For this reason, germ-line gene therapy is subject to rigorous ethical and regulatory scrutiny; it is currently considered highly controversial and is primarily restricted for use in research [28,29].

Somatic Gene Therapy

In contrast, somatic gene therapy involves modifying the genetic material within somatic cells, which are non-reproductive cells found throughout the body. When therapeutic genes are introduced into somatic cells, the resulting changes and associated effects are limited to the patient undergoing treatment. These genetic modifications are not inherited and do not impact the patient's descendants. Somatic gene therapy has taken the forefront in gene therapy research and clinical applications, where it finds application in treating a diverse array of genetic and acquired disorders. This includes conditions such as cystic fibrosis, severe combined immunodeficiency (SCID), and multiple cancer types. The approach is considered safer and more ethically straightforward than germ-line gene therapy because it does not have implications for future generations. Somatic gene therapy has shown promising results and has been used successfully to treat certain diseases. However, like all medical interventions, it continues to be subject to rigorous testing, safety evaluations, and regulatory oversight to ensure its effectiveness and safety for patients [28,29].

Gene delivery

An innovative medical approach is embraced by gene therapy, wherein genetic material is transferred to specific target cells, enabling them to produce essential proteins for combating various diseases [4,30,31]. It is particularly effective for treating disorders resulting from single-point mutations, making it a promising medical avenue [32]. The choice of gene transfer method in this therapeutic approach depends on several factors, including the necessary period for releasing the protein and the physical characteristics of the target tissue. A frequently used process for gene delivery is viruses as carriers or vectors. These viruses are genetically modified to carry therapeutic genes into the patient's target cells. Several types of viruses have been adapted for this purpose. Retroviruses, such as HIV, can produce double-stranded DNA copies from their RNA genomes, which can subsequently be integrated into the chromosomes of host cells. Adenoviruses, with double-stranded DNA genomes, are notorious for causing respiratory, intestinal, and eye infections in humans, including the common cold. Adeno-associated viruses are small, single-stranded DNA viruses that can insert their genetic material at specific locations on chromosome 19 [33]. Herpes simplex viruses are double-stranded DNA viruses, primarily infecting neurons, including herpes simplex type 1, an ordinary human pathogen associated with conditions such as cold sores [33].

Apart from viral vectors, non-viral methods are also employed in gene therapy, known as transfection. The direct DNA introduction method directly inserts therapeutic DNA into the target cells. While it is a relatively straightforward approach, it has limitations regarding the types of tissues it can effectively target and the substantial amounts of DNA required [31-33]. Liposome-mediated delivery is an alternative non-viral approach that employs liposomes in artificial lipid spheres containing an aqueous core. These liposomes carry therapeutic DNA and can traverse the target cell's membrane, facilitating the delivery of genetic material [33]. This method expands the possibilities of gene therapy applications by broadening the range of tissues that can be effectively targeted. Two distinct ways come into play when contemplating gene transfer for clinical applications. In the case of in vivo gene transfer, the foreign gene is introduced directly into the patient's body through viral or nonviral techniques. On the other hand, ex vivo gene transfer entails the introduction of a foreign gene into cells extracted from a tissue biopsy, which is performed outside the patient's body. These genetically modified cells are then transplanted back into the patient, completing the therapeutic process [34].

Applications in dentistry

**Figure 1:**
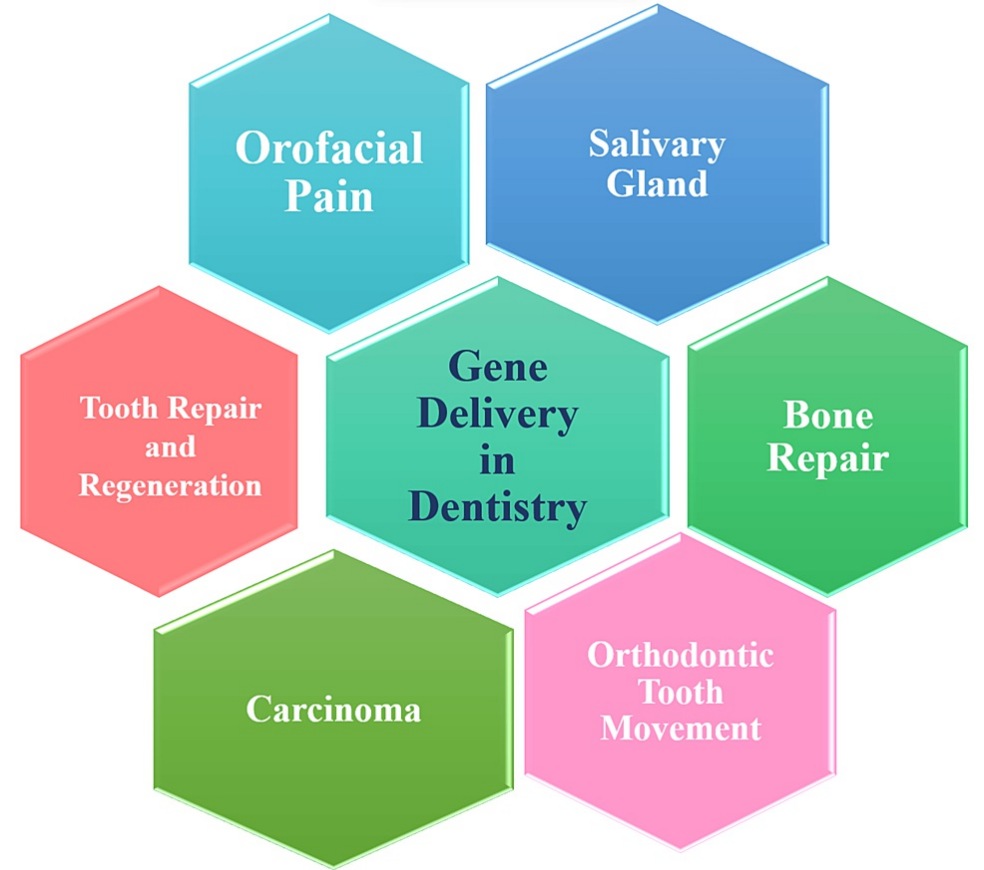
Applications of gene delivery in dentistry. Image Credit: Author Unnati Shirbhate

Remarkable progress has been achieved in unlocking numerous applications of gene therapy within the realm of dentistry, as vividly illustrated below in Figure 1. 

Bone Repair

Bones exhibit an impressive regenerative and reparative potential, distinguishing them from other dental hard tissues such as enamel and dentin [35,36]. In dentistry, ex vivo methods have introduced genes that encode bone morphogenetic proteins (BMPs) [37]. BMPs are widely recognised as catalysts for inducing ectopic and orthotopic bone formation. In most instances, bone fractures and injuries can heal without resulting in unsightly scars. However, bone healing and remodelling can become challenging when dealing with pathological fractures or substantial bone deformities [38-40].

A potent mitogen, platelet-derived growth factor (PDGF), is pivotal during wound healing. PDGF exerts antiapoptotic effects and significantly impacts critical aspects of the healing process, such as cell migration, proliferation, and the synthesis of the extracellular matrix. The growth arrest (GAS) gene can inhibit PDGF activity. To overcome this inhibition, the development of a bioactive PDGF gene has played a crucial role in bypassing the inhibitory effects of the GAS Gene, ultimately promoting effective wound healing. [41]. Bone sialoprotein, in tandem with the core-binding factor subunit alpha-1 (CBFA1) gene, actively contributes to the intricate process of bone healing by playing a pivotal role in cell differentiation and regulating gene expression related to bone sialoprotein during bone repair and regeneration [42].

Salivary Glands

In salivary gland gene therapy, retroductal cannulation of primary excretory ducts is crucial for producing therapeutic proteins and molecules. Various genes, including those related to hormones and antibacterial agents, are utilised [43]. Dry eyes and mouth characterise Sjogren's syndrome (SS), an autoimmune condition that is often coexistent with other immune disorders like lupus and rheumatoid arthritis. Innovative treatments are being developed, including gene-based therapies that regulate immune responses. These therapies may involve anti-inflammatory cytokines like IL-10 or vasoactive intestinal peptide, which can reduce proinflammatory cytokines and help maintain salivary glycoprotein function [44].

Carcinomas

A group of cancers affecting the oral cavity, paranasal sinuses, larynx, pharynx, and head and neck skin is encompassed by squamous cell carcinoma of the head and neck (SCCHN), which stands as the sixth most prevalent cancer globally [38]. In the pursuit of effective treatments for squamous cell carcinoma, a unique gene therapy strategy has been extensively explored, one that selectively amplifies within tumour cells and subsequently lyses them. A significant stride in treating malignancies with deficient tumour protein p53 (TPp53) activity is the development of ONYX-015, which is an adenovirus engineered with a specific deletion of the early 1B 55-kDa protein (E1B 55kD) gene. Patients dealing with recurrent or refractory squamous cell carcinoma have undergone secure administration of ONYX-015 via intratumoral injection. However, when utilised as an individual gene therapy, the evidence of its antitumour efficacy has been somewhat restricted [45]. Nevertheless, a remarkable stride was achieved through a significant breakthrough when a phase II study effectively combined intratumoral injection of ONYX-015 with cisplatin and 5-fluorouracil for patients with recurrent SCCHN. This innovative approach resulted in notable objective responses, including a substantial percentage of complete responses. Of utmost significance, none of the responsive tumours exhibited progression at the six-month milestone. Subsequent examination of tumour samples post-treatment unveiled tumour-selective viral replication and the induction of necrosis. Intriguingly, the study did not reveal an apparent correlation between p53 mutations in the tumour and the clinical outcome, underscoring the necessity to reduce bystander interactions and advance the development of systemic administration agents [38,45].

Orofacial Pain

Orofacial discomfort covers pain in the face, head, and neck tissues. Dealing with orofacial pain syndromes, including chronic cases, is complex and unclear. These syndromes vary from those with clear causes to idiopathic ones. Untreated acute diseases can lead to chronic pain in around 20% of cases. The management of pain typically includes the use of pain-relieving medications and calming agents. Gene therapy offers promise in treating chronic pain and reducing reliance on drugs with adverse effects. While mainly studied in animal models for now, gene therapy, particularly with advanced vector systems, could become more effective for pain syndromes like trigeminal neuralgia and temporomandibular joint diseases [46].

Tooth Repair and Regeneration

The dental pulp has long been recognised for its remarkable reparative and regenerative capabilities. Dental pulp cells have the unique capacity to undergo terminal differentiation, giving rise to cells that closely resemble odontoblasts, which play a pivotal role in the generation of reparative dentine. Gene therapy experiments have revealed that when pulp cells are transfected with growth/differentiation factor 11, their odontogenic differentiation capabilities are notably enhanced. Researchers have explored various methods to stimulate the differentiation of pulp cells into odontoblast-like cells, which include the application of synthetic glucocorticoid dexamethasone and growth factors (GFs), such as BMP2 [47]. In the context of in vivo gene therapy, the direct application of genes that promote dentine development to exposed tooth pulp has shown promising results in enhancing the recovery potential of tissues like the dentine pulp complex. Moreover, dental pulp stem cells hold the potential to serve as a novel and versatile cell population for a range of applications, including heart, bone, muscle, brain, and tooth repair and regeneration, as demonstrated by in vivo experiments in animals. The successful first clinical application of dental pulp stem cells for alveolar bone restoration in a patient was achieved [48].

Orthodontic Tooth Movement

Alveolar corticotomy surgery, while effective in speeding up orthodontic treatment, has drawbacks, such as high morbidity rates. The biological processes of bone resorption and apposition play a crucial role in tooth movement (TM), regulated by receptor activator of nuclear factor kappa ligand (RANKL) and osteoprotegerin (OPG). Corticotomy-assisted malocclusion treatment relies on the regional acceleratory phenomenon to expedite bone remodelling, reducing TM time. We propose that sustained RANKL overexpression can enhance osteoclast activity and bone resorption, accelerating TM throughout treatment, not just at the beginning. A promising strategy for shifting ankylosed teeth and shortening orthodontic therapy is the local transfer of the RANKL gene. In contrast, tooth displacement is slowed by about 50% over 21 days with the local transfer of the OPG gene [4]. This groundbreaking approach promises to revolutionise orthodontic care, shorten treatment, and improve outcomes. Additionally, gene therapy can alleviate orthodontic TM discomfort, with further research paving the way for new therapeutic solutions.

Applications in periodontics

Periodontal Vaccination

The introduction of plasmid DNA containing the Porphyromonas gingivalis (Pg) fimbrial gene into a mouse's salivary gland prompts the localised production of fimbrial protein. As a result, specific serum and salivary IgG and IgA antibodies are generated, effectively countering Pg and preventing plaque growth. Moreover, studies conducted in rats have demonstrated the effectiveness of vaccination using genetically engineered Streptococcus gordonii vectors expressing the Pg fimbrial antigen in preventing Pg-associated periodontitis. Another significant development is hemagglutinin, a crucial element contributing to Pg lethality. When rats infected with Pg are exposed to recombinant hemagglutinin B (rHag B), an immune response is initiated, leading to the production of protective serum IgG antibodies and the synthesis of IL-2, IL-10, and IL-4; this immune reaction effectively safeguards against Pg-induced bone loss [49]. 

Biofilm Antibiotic Resistance

Research has shown that bacteria capable of forming biofilms exhibit remarkable resilience to antibiotics, with resistance levels reaching up to a thousand times that of their non-biofilm-forming counterparts. This heightened resistance poses a significant challenge in terms of control. In a recent study, the gene ndvB was found in the Pseudomonas aeruginosa RA14 strain. This gene encodes the glycosyltransferase required for the biosynthesis of periplasmic glucans, emphasising a potential target for subsequent research and intervention [49].

Electroporation for Alveolar Bone Remodelling

Electroporation, also known as electropermeabilisation, involves subjecting cells to an electrical field to improve the permeability of the cell membrane. This process, known as electrotransfer, may make it possible to inject chemicals, drugs, electrode arrays, or DNA into the cell. When nonviral BMP-2/7 gene expression vector and in vivo electroporation are used in gene therapy for alveolar bone regeneration, the potential for alveolar bone regeneration in the targeted area can be increased for up to three weeks. The periodontal tissue undergoes a robust regenerative process involving synthesising various bioactive molecules in response to mechanical strain and inflammation factors. Notably, successful and predictable alveolar bone regeneration has been achieved through in vivo transfer of the β-galactosidase or LacZ gene. This gene encodes a multitude of molecules involved in tissue remodelling. The transfer is facilitated by employing plasmid DNA as a vector and transient transfection using electric impulses to introduce the gene into the periodontal ligament (PDL) cells [50].

Tight Adherence Gene for Control of Periodontal Disease Progression

At the outset of localised aggressive periodontitis, a critical step involves the infiltration of target tissues by a periodontal pathogen, Aggregatibacter actinomycetemcomitans, which establishes its virulence through a mechanism known as "tight adherence" for attachment. Researchers have identified a mutant strain lacking the "tight adherence gene." This groundbreaking discovery holds the potential to significantly impact the progression of periodontal disease by hindering the conscription and pathogenic activity of Aggregatibacter actinomycetemcomitans [50].

*Antimicrobial Gene Therapy to Control Disease Progression* 

One method of underpinning the host's defence mechanism against infections involves introducing genes that encode antimicrobial peptides or proteins into host cells. In a significant study, raising the defensin-2 (HBD-2) gene into host cells in vivo, using a retroviral vector, substantially improved the host's antimicrobial defences. Additionally, a promising avenue for treating periodontal disease involves designer drug therapy. This approach focuses on identifying the genes responsible for standard development and devising therapeutic interventions targeting the identified gene abnormalities. Such designer therapies offer a potentially safer alternative to current pharmaceutical treatments, as they exclusively address the known genetic abnormalities determined through genetic research [51].

Limitations

Gene therapy, a pioneering field continuously advancing, presents challenges and risks akin to any medical intervention. Below are some of the significant concerns related to gene therapy. Only temporary relief is often provided by gene therapy. For benefits to be sustained, the therapeutic DNA launched into target cells must remain functional, and these cells must exhibit stability and longevity. Key challenges encompass the integration of therapeutic DNA into the genome and the speedy distribution of numerous cell types, potentially limiting long-term effectiveness [2,5]. When foreign genetic material is introduced, the immune system can perceive it as a threat and mount a response, potentially reducing the therapy's effectiveness. This immune reaction can hinder repeat gene therapy attempts in patients [5].

Viral Vector Concerns

Viruses are commonly used as carriers in gene therapy but can pose various issues. These include potential toxicity problems, eliciting immune and inflammatory responses, and encountering gene control and targeting difficulties. Additionally, concerns arise regarding the potential for a viral vector, once introduced into the patient, to potentially regain its ability to induce disease [2,5]. Heart disease, high blood pressure, Alzheimer's disease, arthritis, and diabetes, among other common conditions, often result from the collective effects of variations in multiple genes. Gene therapy most effectively treats single-gene disorders, posing a significant challenge in addressing multigenic or multifactorial diseases [2]. A considerable concern lies in the potential integration of therapeutic DNA into an incorrect genomic location. If, for instance, it integrates into a tumour suppressor gene, it could initiate tumour development. Clinical trials have documented this risk for specific genetic disorders, underscoring the necessity for precise targeting [2]. Gene therapy can be expensive, and the price may limit its accessibility to a broader population. This cost includes research, development, and clinical implementation. Ethical considerations surrounding gene therapy, such as questions about consent, genetic modification, and long-term consequences, must be addressed [5].

Future aspect

The future of gene therapy in dentistry and periodontics holds great promise, offering innovative approaches to treat various oral and periodontal conditions. Here are some potential future perspectives for gene therapy in these fields. Gene therapy has the potential to usher in an era of personalised dental care. Genetic profiling could help identify individuals at a higher risk of dental and periodontal issues. Tailored gene therapies can then be developed to address specific genetic factors contributing to these conditions. Gene-based treatments will likely be pivotal in regenerating damaged or lost dental and periodontal tissues. This could include regrowing teeth, restoring damaged periodontal ligaments, and regenerating alveolar bone, offering more effective and long-lasting solutions. Gene therapy may revolutionise pain management strategies in dentistry. Customised approaches to mitigate discomfort during procedures, such as orthodontic tooth movement, could minimise patient distress and improve treatment outcomes. As our understanding of the genetic underpinnings of multifactorial dental and periodontal diseases grows, gene therapy could target multiple genetic factors simultaneously, offering more comprehensive and effective treatments.

Gene therapy might lead to the development of highly targeted antimicrobial treatments for periodontal diseases. Enhancing the body's natural defences or introducing genes that combat specific pathogens could offer more precise and less disruptive alternatives to traditional antibiotics. Gene therapy research may uncover new therapeutic targets within the oral cavity, leading to the growth of innovative treatments for conditions that are currently challenging to manage. Gene therapy can accelerate orthodontic tooth movement and reduce treatment times. This innovation may significantly enhance the patient experience and expand the use of orthodontic treatments. Future developments will focus on improving the safety and longevity of gene therapies, minimising potential risks associated with integrating foreign genetic material and ensuring sustained benefits. Unlocking the full potential of gene therapy in dentistry and periodontics necessitates ongoing research, clinical trials, and collaboration between geneticists, dentists, and periodontists. These future perspectives promise more effective, personalised, and patient-friendly oral and periodontal health solutions.

## Conclusions

In conclusion, gene therapy in dentistry and periodontics holds significant promise for revolutionising the treatment of various oral and periodontal conditions. From regenerating dental tissues to combating oral cancers, gene therapy has showcased its potential in preclinical and clinical studies. Despite challenges like ensuring long-term gene functionality and potential immune responses, ongoing research and advancements in gene delivery mechanisms provide hope for the progress of successful gene-based treatments in the field. The future of dentistry and periodontics may witness innovative therapeutic approaches that alleviate patient discomfort, reduce treatment times, and enhance treatment outcomes. However, it is imperative that safety, ethical considerations, and thorough clinical testing continue to guide the evolution of gene therapy in these disciplines, ensuring that patients can reap the benefits of this cutting-edge technology while minimising associated risks.
